# A Novel *In Vivo* Model to Study Impaired Tissue Regeneration Mediated by Cigarette Smoke

**DOI:** 10.1038/s41598-018-28687-1

**Published:** 2018-07-19

**Authors:** Marjorie Alvarez, Myra N. Chávez, Miguel Miranda, Geraldine Aedo, Miguel L. Allende, José T. Egaña

**Affiliations:** 1 FONDAP Center for Genome Regulation, Facultad de Ciencias, Universidad de Chile, Santiago, Chile; 2 Advanced Center for Chronic Disease (ACCDiS) & Center for Molecular Studies of the Cell (CEMC), Facultad de Ciencias Químicas y Farmacéuticas & Facultad de Medicina, Universidad de Chile, Santiago, Chile; 3 Institute for Biological and Medical Engineering, Schools of Engineering, Medicine and Biological Sciences, Pontifícia Universidad Católica de Chile, Santiago, Chile

## Abstract

Cigarette smoke is associated with several pathologies including chronic respiratory diseases and cancer. In addition, exposure to cigarette smoke is correlated with impaired wound healing, where a significant decrease in the regenerative capacity of smokers is well documented and broadly considered a negative risk factor after trauma or surgery. So far, some *in vitro* and *in vivo* models have been described to study how exposure to cigarette smoke diminishes the regenerative potential in different organisms. However, although useful, many of these models are difficult and expensive to implement and do not allow high-throughput screening approaches. In order to establish a reliable and accessible model, we have evaluated the effects of cigarette smoke extract (CSE) on zebrafish development and regeneration. In this work, zebrafish embryos and larvae were exposed to low doses of aqueous CSE showing severe developmental abnormalities in a dose-dependent manner. Furthermore, when adult zebrafish were subjected to caudal fin amputation, we observed a significant decrease in the regenerative capacity of animals exposed to CSE. The effect was exacerbated in male and aged fish compared to female or young organisms. The establishment of a zebrafish model to assess the consequences of cigarette smoke and its effects on animal physiology could provide a new tool to study the underlying mechanisms involved in impaired tissue regeneration, and aid the development of novel approaches to treat complications associated with cigarette smoke toxicity.

## Introduction

Tobacco smoking is a worldwide epidemic that represents the second major cause of death and the fourth most common risk factor for disease. According to the World Health Organization, “it is the only legal drug that kills up to half of its users when used exactly as intended by manufacturers”, representing the major preventable cause of persistent disability and death in developed countries^1,2^. Smoking is broadly associated with several diseases, including chronic obstructive lung disease, lung cancer, coronary heart disease, cerebrovascular disease and cancer^3^. Furthermore, the negative effects of tobacco smoking on tissue regeneration have been singled out as a major issue of concern in public health and clinical practice. Several processes relevant to wound-healing, such as white blood cell recruitment, susceptibility to bacterial infection, fibroblast migration and proliferation, wound contraction and re-epithelialization, as well as extracellular matrix production, have all been reported to be aggravated in smokers^4,5^. Also, exposure to both first- and second-hand cigarette smoke, has been associated with impaired wound healing, and is correlated with worse surgery outcomes across all surgical specialties^6^. Additionally, the habit of smoking has been shown to increase the likelihood of surgical problems, as well as many postoperative complications, such as a higher incidence of infection and wound dehiscence after plastic surgery procedures^7^. Chronic exposure to cigarette smoke has been reported to have adverse effects on bone fracture repair and ligament healing that include heightened pain, longer hospitalization periods, and increased rate of hospital readmission^8–10^. Furthermore, smoking is considered a risk factor for non-healing and major amputation diagnosis in patients undergoing diabetic foot ulcer surgical treatment^11^, while a negative impact in number, size and overall healing of pressure injuries have also been reported^12^. On the other hand, smoking cessation is considered to be key for optimal health care delivery for patients with critical limb ischemia^13^ and clinical procedures involving the musculoskeletal system in general^14^.

In order to develop new therapeutic approaches and study the mechanisms behind the detrimental effects of cigarette smoking on development, chronic diseases as well as wound healing, several *in vitro* and *in vivo* models have been established. For instance, we, as well as others have shown that cigarette smoke extract (CSE) has detrimental effects in different cell culture models. CSE is also known to impair mesenchymal stem cell migration, as well as their osteogenic and chondrogenic differentiation potential^15,16^. Furthermore, CSE-induced damaged has been demonstrated in osteoblasts^17,18^, endothelial cells^19–21^, and fibroblasts^22,23^. Although these models may provide important information at a molecular level, extrapolation of the results is limited, especially when it comes to understanding the response of whole organisms.

*In vivo* murine models, on the other hand, have been reported to replicate the damage in bone integrity and the compromised bone regeneration outcome in tobacco consumers as well as in people exposed to secondhand smoke^24–26^. These models have also been used to study the effect of exposure to cigarette total particulate matter (TPM) in skin wound healing^27^, and to address the damage induced by cigarette smoke on the olfactory epithelium, the impairment of nasal mucosa healing and the recovery of olfactory receptor neurons^28,29^. Finally, the chicken chorioallantoic membrane assay has been used to study the atrophies induced by cigarette smoke condensates and TPM on the integrity of the microvasculature^30^.

However, most of the above described models are either difficult, laborious, or expensive to implement, and fail to replicate or distinguish the complex effects of acute versus chronic cigarette smoke exposure in a whole organism. Additionally, some of the reported studies only use nicotine or TPM, which do not represent the mixture of more than 4700 different toxic molecules present in cigarette smoke^31–33^ therefore limiting the scope of their findings. In this regard, the zebrafish has become a powerful model for toxicological studies given its many advantages. Among others, its small size at the embryonic and larval stages, large number of offspring, optical transparency, permeability, availability of transgenic lines, and rapid development in an aqueous environment. Additionally, both, its cardiovascular and innate immune systems are homologous to human, which makes them ideal to study cellular interactions *in vivo*^34^. To date, some studies have already addressed the effects of cigarette smoke extracts on larval development, and compared them to the effects of nicotine alone^35,36^. Furthermore, the toxicity of other alternative forms of nicotine intake, such as electronic cigarettes or snuffing, and also passive smoking through side stream smoke and third-hand smoke have been addressed using the zebrafish embryo and larvae as a models^37–39^. Yet, the effect of cigarette smoke exposure on zebrafish regeneration remains to be addressed.

The caudal fin of the zebrafish represents a unique and tractable model to study complex tissue regeneration. After transection of a defined segment of the caudal fin, the fin is capable to faithfully regenerate within 10–14 days. Bone, skin, blood vessels, nerves and sensory organs all return to their normal appearance and function as a result of epimorphic regeneration^40^. This appendage has the additional advantage of being thin and therefore transparent enough to allow the visualization of cell-cell interactions and tissues in the living animal, a feature powerfully complemented by the availability of transgenic strains expressing fluorescent proteins in diverse tissue types and organs. In this work, we decided to evaluate whether the zebrafish model could be established to study the toxic effects of fresh whole cigarette smoke extracts during embryonic and larval development, as well as a model for smoke-dependent impaired tissue regeneration in adult tissues.

## Material and Methods

### Zebrafish breeding and animal housing

Zebrafish (*Danio rerio*) embryos from the wild type AB strain, or the transgenic strain *Tg*(fli1a:EGFP)y1^41^ were obtained from our breeding colony. All embryos were collected by natural spawning and raised at 28.5 °C in E3 medium (5 mM NaCl, 0.17 mM KCl, 0.33 mM CaCl2, 0.3 mM MgSO4, and 0.1% methylene blue, adjusted to pH 7.0) in Petri dishes. Egg water was changed daily. Embryonic and larval ages are expressed in hours or days post-fertilization (hpf or dpf). Adult zebrafish were maintained under monitored water conditions (28.5 °C, pH 7–7.3, 600–800 μS), in 14:10 light-dark cycle conditions, at a density of 3–4 fish/1 L, and daily fed with *Artemia salina*, dry flake food (Tetra, Spectrum Brands, USA) and dry particularized food (Gemma, Skretting, Norway). Animals were anesthetized with MS-222 (Tricaine, A5040, Sigma-Aldrich, MO, USA) before each experiment. All procedures complied with the “Guidelines for the Use of Fishes in Research Use” of the American Fisheries Society (Use of Fishes in Research- Committee, a joint committee of the American Fisheries Society, the American Institute of Fishery Research Biologists, and the American Society of Ichthyologists and Herpetologists. 2014. Guidelines for the use of fishes in research. American Fisheries Society, Bethesda, Maryland. www.fisheries.org), and were approved by the Animal Ethics Committee of the University of Chile.

### Generation of cigarette smoke extracts

Cigarette smoke extracts (CSE) were obtained as described before^15^. Briefly, CSE was generated by bubbling the combustion of three cigarettes of a popular commercial brand with their filter removed, into 25 ml egg-water using a 125 mL gas washing bottle with two exits, one connected to a vacuum pump and the other one especially adapted to hold the cigarette. Suction is accomplished through the negative pressure induced by the vacuum pump, and regulated to achieve a standardized smoking time of 3-minutes per cigarette. This CSE-stock solution was then diluted to the described concentrations in each experiment (v/v) before administration to the zebrafish. Both CSE-stock solution and dilutions were always freshly prepared before exposure.

### Exposure of larva zebrafish to CSE and determination of LC50

3–5 hpf embryos were incubated with different concentrations of CSE for 96 hours at 28.5 °C. Besides survival, following parameters were chosen to evaluate the development of the embryos:

- edema formation: scored by presence/absence of edema.
- normal heart beat: less than 1 beat/sec was considered abnormal; normal embryonic heart rate is 120–180 beats per minute^42^.
- blood circulation: complete lack of circulation of erythrocytes observed in the posterior part of the body.
- head-trunk angle: less than 30° compared to the respective angle according to the developmental state was considered abnormal^43^.
- somite development: a difference ≥2 in the number of somite according to the developmental state was considered abnormal^43^.
- otic vesicles: no formation of the elliptic otic vesicle (24 hpf), no distinguishable otholiths (48 hpf), or abnormal shape of the otic vesicle (72–96 hpf) were considered abnormalities.
- eyes: abnormal phenotype considered a decreased size of the eyes compared to non-treated controls or non-uniform eye pigmentation.
- pigmentation: absence of black melanocytes over the yolk, head or caudal fin (48 hpf) or along the notochord (72–96 hpf) was considered abnormal.
- swim bladder: no development (inflation) of the swim bladder or incapability to swim at 96 hpf was considered abnormal.
- spontaneous movement inside the chorion: no twisting behavior within one minute at 24 hpf was considered abnormal.
- swimming response to mechanical stimulation: 48–96 hpf larvae were touched with a pipette tip, slow or no response to the stimulus was considered abnormal.
- eclosion: “free larvae”, the percentage of larvae outside their chorion was calculated at 48–96 hpf.

Vascular development was evaluated using transgenic Tg(fli1a:EGFP)^y1^ embryos. The formation and connection of the five intersegmental blood vessels (ISVs) anterior to where the extension of the yolk connects to the urogenital opening were observed as a practical measure to assess correct vascular development (angiogenesis) after 24 hours of exposure to CSE. In order to determine the median lethal concentration of CSE (LC50), the number of dead embryos, e.g. when neither heart beat nor circulation were detected, was estimated following the OCDE fish embryo toxicity test (zFET) protocol and data were then subjected to the Probit analysis^44^.

### Exposure of adult zebrafish to CSE

Zebrafish, of the indicated ages and sex, were selected and randomly divided into groups. CSE was generated as described above, and CSE-dilutions were prepared in fish water as stated in the experiment description. Experimental fish were exposed to different concentrations of CSE while held individually in separate compartments at a minimum of 400 ml water per fish. Fish were fed as usual, and fish water was changed daily maintaining the respective CSE-concentration in the water for the duration of the experiment.

### Caudal fin amputation procedure

Wild type and Tg(fli1a:EGFP)^y1^ transgenic adult fish were maintained in 0.25% CSE- fish water for 10 days, with daily water changes as described above. Caudal fin amputation was performed on anesthetized animals using razor blades as described before^45^. Treatment with 0.25% CSE-fish water continued for 10 days post-amputation, while fish water was exchanged accordingly in the control group. Zebrafish regarded as “young” (N ≥ 22) were 6–12 months old, while “aged” fish (N ≥ 10) were 18–24 months old and chosen randomly in regard to gender. For experiments comparing female (N ≥ 15) and male fish (N ≥ 15), individuals of the same age were used.

### Zebrafish visualization

Zebrafish embryos and larvae were anesthetized and evaluated in regards to the parameters described above using a dissecting microscope. For bright field imaging zebrafish embryos and larvae were fixed in 4% paraformaldehyde, and mounted in 1% low-melt point agarose (BM-0133, Winkler, Chile). For confocal imaging of the GFP-labelled fish vasculature at 24 hpf, transgenic larvae were dechorionated, anaesthetized and mounted. Then, Z-stacks were taken at a distance of 5 μm between layers (Zeiss LSM 510 confocal microscope, Plan-Neofluar 25×/0.8 Imm corr DIC, Carl Zeiss AG, Germany) from which Z-projections were made and merged to create the final image using the ImageJ software^46^. Wild type and Tg(fli1a:EGFP)^y1^ transgenic adult fish were anesthetized for caudal fin imaging, pictures were taken under a fluorescent stereoscope (Olympus MVX10, Olympus Corporation, Japan) and returned to their tanks for recovery.

### Quantitative evaluation of fin regeneration and re-vascularization

The caudal fin regeneration progress was monitored by imaging the re-growing fin tissue every 2 days under a stereoscope (Olympus MVX10, Olympus Corporation, Japan). Fin regeneration was quantified by digitally measuring the area from these images using ImageJ^46^. In order to compare the regeneration potential, we calculated the “regeneration speed” based on the slope obtained by linear regression analysis of the fin area measurements. To determine the degree of neovascularization within the regenerating caudal fin tissue, a ratio of the area covered by the GFP-labelled fish vasculature and the complete regenerated fin area was created. Area measurements were obtained by blinded image analysis using the software ImageJ^46^.

### Statistical analysis

Results were gathered from at least 3 independent experiments. Data is expressed as mean ± SD. To calculate statistical relevance, two-tailed student’s t-test or one-way ANOVA followed by Dunnett’s test were used to compare differences between experimental and control groups according to the experiment. Difference among means was considered significant when p ≤ 0.05.

### Data availability

The datasets generated during the current study are available from the corresponding author on reasonable request.

## Results

**Figure 1:**
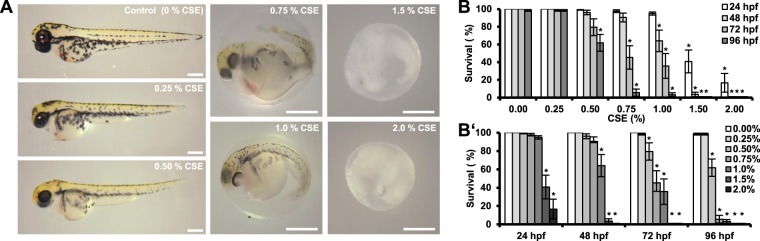
Evaluation of cigarette smoke toxicity in zebrafish. 3–5 hpf zebrafish embryos were subjected to increasing concentrations of CSE for 96 hours. Representative pictures of the larvae after 48 hours of treatment are shown (**A**). Zebrafish survival was recorded daily, showing a dose-dependent lethal effect of continuous CSE-exposure in zebrafish larvae (**B**,B’). Results were acquired from three independent experiments, using a total of N ≥ 120 embryos for each condition. *p < 0.05. Scale bar represents 200 μm.

In order to evaluate whether zebrafish represents a viable model to study the toxicity of aqueous cigarette smoke extracts (CSE), 3–5 hpf embryos were incubated for 96 hours with different concentrations of CSE. CSE toxicity showed to be both concentration- and exposure time-dependent, and obvious effects were observed by light microscopy, ranging from decreased pigmentation at low concentrations to death at higher doses (Fig. 1A). While embryos were able to survive in E3 containing 0.25% CSE during the entire experiment, concentrations above 1% CSE were lethal to a significant percentage of the animals already after 24 hours of exposure (Fig. 1,B and B’). Meanwhile exposure to 0.5% CSE resulted in a significant mortality of the larvae after 72 hours. Based on the FET-Test standard and Probit analysis of the data, we calculated the median lethal concentration of CSE to be 1.47 ± 0.04, 1.09 ± 0.01, 0.79 ± 0.03 and 0.54 ± 0.0 (%, v/v) for 24, 48, 72, and 96 hours of treatment, respectively.

**Figure 2:**
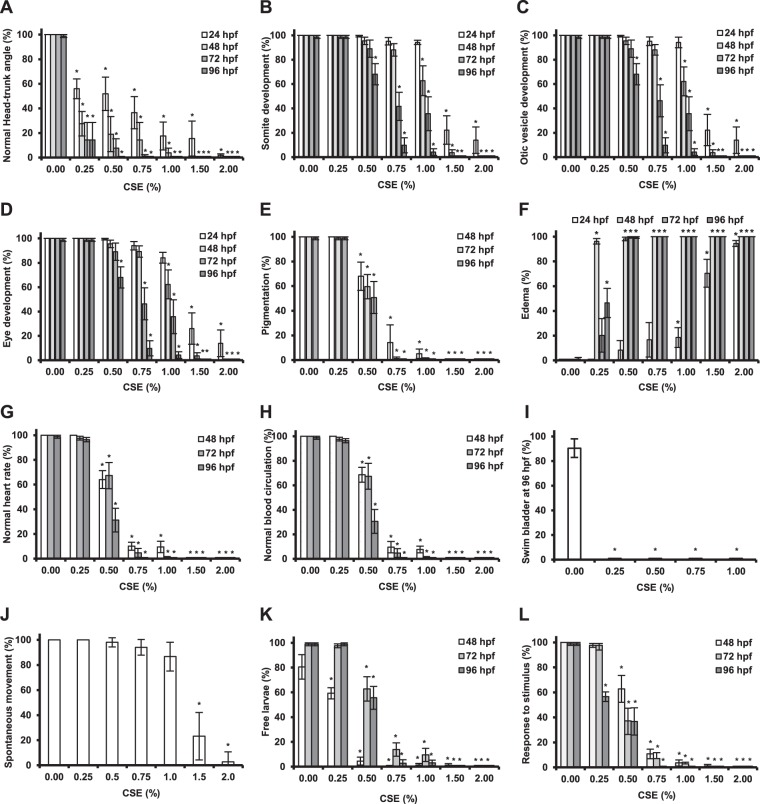
Effects of cigarette smoke on zebrafish embryonic and larval development. The effect of increasing CSE-concentrations on zebrafish development at different embryonic and larval stages was evaluated and compared to a non-treated control (0.00% CSE) and based on the following parameters: normal head-trunk angle (**A**), correct somite development (**B**), normal otic vesicle development (**C**), normal eye development (**D**), normal pigmentation (**E**), heart edema formation (**F**), normal heart rate (**G**), normal blood circulation (**H**), swim bladder inflation (**I**), spontaneous movement inside the chorion (**J**), eclosion (**K**), response to mechanic stimulus (**L**). The percentage of embryos/larvae showing the abnormal phenotype, as described in the materials and methods section, is shown. Results were obtained from three independent experiments, using a total of N ≥ 120 embryos for each condition. *p < 0.05.

Moreover, even low doses of CSE had severe effects on the development of zebrafish embryos and larvae. For instance, exposure to 0.25% CSE already induced a significant developmental delay as seen in the deviation from the normal head-trunk angle in 24 hpf embryos (Fig. 2A). 0.5 % CSE was enough to significantly impair proper somite development (Fig. 2B), formation of otic vessicles (Fig. 2C), eyes (Fig. 2D), and pigmentation (Fig. 2E) in 96 hpf larvae. This concentration also increased the percentage of edema formation by 10% compared to the control embryos at 24 hpf (Fig. 2F), while affecting the larvae heart beat and circulation significantly at 48 hpf (Fig. 2G,H). In addition, swim bladder inflation and eclosion were also affected by 0.25% CSE (Fig. 2I,K), thus seriously compromising the future survival of the larvae. In addition, embryos displayed inability to move spontaneously after exposure to 1.0% CSE for 24 hours (Fig. 2J), while the larvae, which were able to survive to CSE-dilutions over 1%, were not motile at 96 hpf anymore (Fig. 2L).

**Figure 3:**
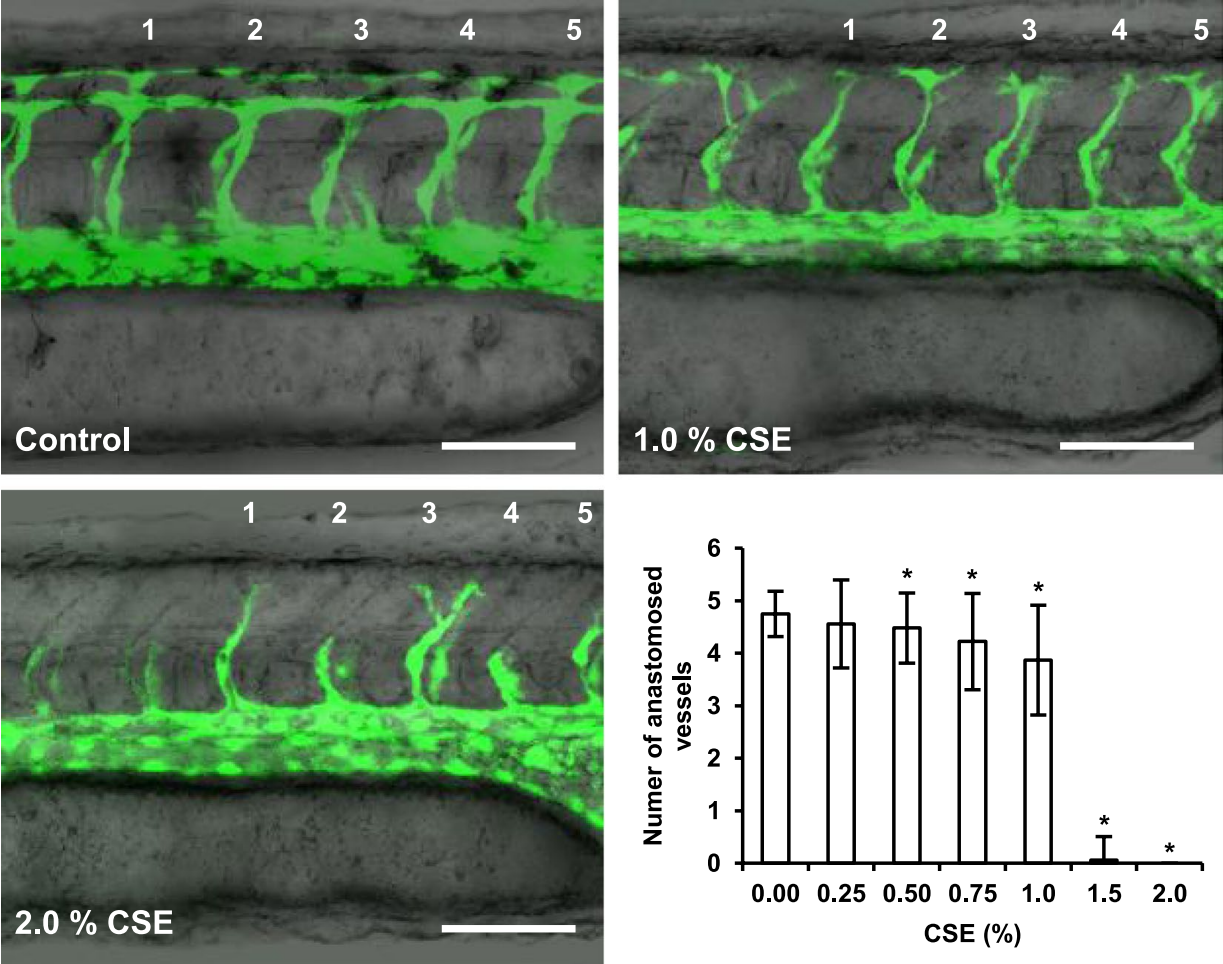
Effect of cigarette smoke on zebrafish vascular development. An impairment of intersegmental vessel (ISVs) formation and extension in one-day old transgenic *Tg*(fli1a:EGFP)^y1^ embryos was observed upon exposure to increasing CSE-concentrations for 24 hours. The number of anastomosed ISVs was quantified as a parameter for vessel maturation for the five anterior vessels proximal to the urogenital opening for each CSE-condition. Results were obtained from three independent experiments, using a total of N ≥ 90 larvae for each condition. *p < 0.05. Scale bars represent 100 μm.

Due its important implications in tissue regeneration, the effect of CSE on vascular development was also studied. 3–5 hpf embryos were exposed to increasing concentrations of CSE for 24 hours, and formation and anastomosis of the five most proximal intersegmental vessels (ISVs) anterior to the urogenital opening were observed for each group in living specimens. With CSE-dilutions above 0.5%, an overall delay in the extension of the ISVs from both aorta and vein was observed, while the number of completely formed and anastomosed ISVs was significantly diminished (Fig. 3). Furthermore, the constitution of vascular network in general seemed to be affected by exposure to CSE, as shown by the thinner and cell-sparse major vessels in the treated groups (Fig. 3, 1–2% CSE).

In an effort to characterize the aqueous CSE used in this study two parameters were evaluated in both the CSE-stock solution and CSE-dilutions, namely, pH and tar-content (Supp. Fig.). The results show a high reproducibility in the experimental setup, taking into account the low standard deviation within the experimental replicates. CSE-dilutions of the extract obtained show a linear correlation with the tar content (Supp. Fig. A), which is consistent with other studies where this method was used as a bench mark^17,18^. On the other hand, they suggest that CSE-toxicity could be linked to a change in the pH, as it decreased significantly with increasing CSE-concentrations (Supp. Fig. B), thus diverging from the near neutrality pH-range physiologically tolerated by the fish.

**Figure 4:**
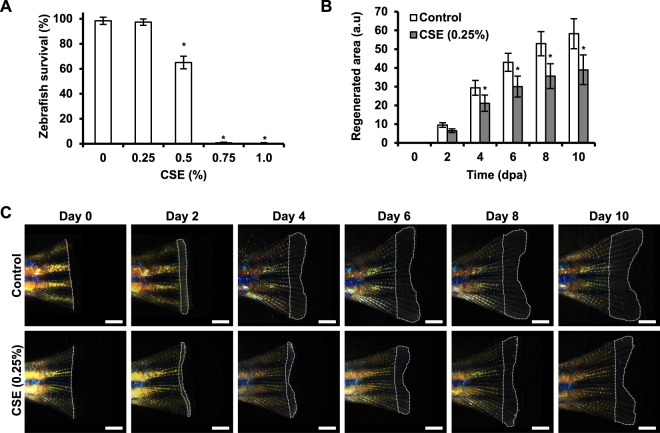
Effect of cigarette smoke on adult zebrafish survival and regeneration potential. The lethal effect of CSE was studied in 6–12 month adult zebrafish exposed to increasing dilutions of cigarette smoke extract for 10 days. Results represent three independent experiments, using a total of N ≥ 15 zebrafish for each condition (**A**). Zebrafish, which underwent tail fin amputation showed diminished regeneration capacity, when exposed to a pre- and post-conditioning treatment with non-lethal concentrations of CSE (0.25%, 20-day treatment in total), as shown by the diminished regenerated fin tissue compared to non-treated condition. Results were obtained from three independent experiments, using a total of N ≥ 25 adult zebrafish for each condition (**B**,**C**). *p < 0.05. Scale bar represents 1 mm.

Once the toxic effects of CSE were well characterized in larvae, we investigated whether similar results could be observed in adult zebrafish. In contrast to larvae, adult zebrafish did not tolerate exposure to CSE concentrations above 0.5%, with only about 60% fish surviving after 10 days (Fig. 4A); exposure to 0.75% CSE caused the death of the fish within 1–2 days (data not shown). We therefore decided to work with sub-lethal CSE-concentrations for the regeneration studies. We observed a high mortality of the fish, if they were exposed to the CSE for the first time right after the fin amputation, so we included a pre-conditioning treatment in our protocol. Thus, the adult fish were pre-conditioned for 10 days by being kept in 0.25% CSE-fish water, with a daily change of water containing freshly prepared CSE-stock solution, while control fish were maintained in similar compartments with a daily water change. Next, control or treated fish were subjected to caudal fin amputation, and the progress of fin regeneration over time was documented by imaging the regenerating tissue every second day (Fig. 4C). After treatment we did not observe tissue necrosis, external signs of inflammation or changes in the general behavior in the experimental group. However, quantification of the regenerating area showed that treatment with CSE significantly decreased the size of the regenerated area from day 4 onward as compared to the control group (Fig. 4B,C).

**Figure 5:**
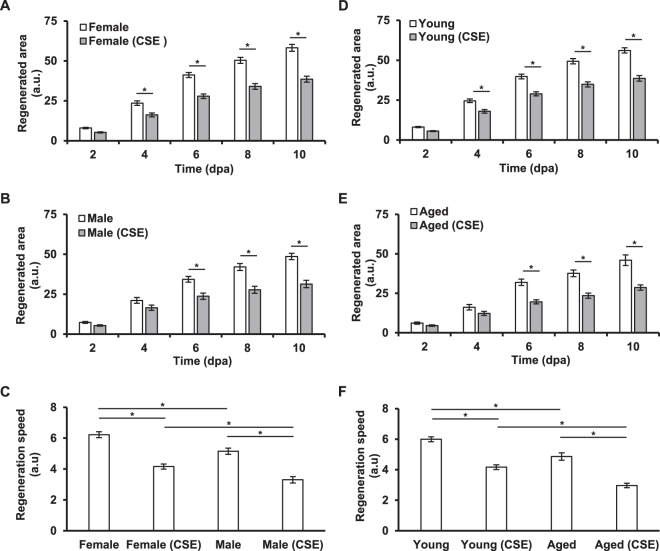
Effect of cigarette smoke in the regeneration potential of zebrafish of different gender and age. Tail fin regeneration outcome was evaluated between control and CSE-treated zebrafish of different ages and gender. While CSE affects regeneration in all conditions, note (in C) that male fish are more susceptible to the effect than female fish, and (in F) that aged fish are more susceptible than young ones. Results represent three independent experiments using a total of N ≥ 10 zebrafish for each condition. *p < 0.05.

We additionally decided to evaluate whether aging and/or sex could influence the effect of CSE in regeneration of the fish caudal fin. For this, using the same experimental protocol as above, the regeneration outcome of female and male zebrafish as well young and aged fish were compared (Fig. 5A–F). As expected, all experiments showed a significantly impaired regeneration outcome in CSE-exposed zebrafish compared to their respective control group. However, our results showed that both, male (Fig. 5C) and aged (Fig. 5F) zebrafish were more susceptible to the regeneration impairment caused by CSE exposure than their counterparts, considering their “regeneration speed”, which was calculated to address their potential to regrow lost tissue.

**Figure 6:**
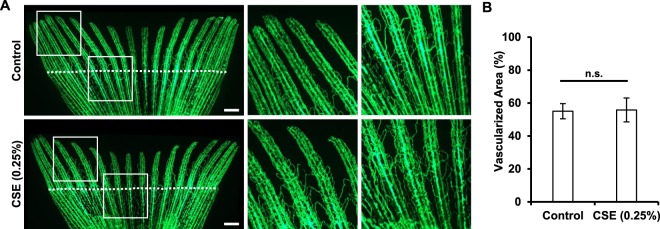
Effect of cigarette smoke extract in adult zebrafish vascular regeneration. Adult *Tg*(fli1a:EGFP)^y1^ zebrafish were exposed to a 0.25% dilution of CSE for ten days before, and 10 days after tail fin amputation. The rate of revascularization was calculated using the signal of the fin fluorescent endothelial cells and normalized to the total regenerated area. While exposure to CSE limited fin regrowth in the treated group, re-vascularization of the fin was not affected. Results were obtained from three independent experiments using a total of N ≥ 20 zebrafish for each condition. *p < 0.05. Scale bars represent 1 mm.

Searching for potential mechanisms involved in the impaired regeneration observed in adult fish and knowing that CSE affects vascular development in embryos (Fig. 3), we analyzed the effects of CSE on the revascularization process that occurs after fin amputation. Surprisingly, we found that vascular density in the regenerating area was not affected by the presence CSE, when compared to the control group (Fig. 6). This finding suggests that this mechanism in particular is not being affected by the CSE treatment in the course of the regeneration process.

## Discussion

It has been estimated that tobacco use is currently responsible for the death of about six million people worldwide every year and, by the year 2030, smoking will become the major source of death and disability, with the number increasing to 10 million deaths per year^1,2^. Furthermore, given that there are over 1.1 billion people smoking tobacco at the present day, this will become an even major issue in the clinical practice and will cost hundreds of billions to health care systems due postoperative complications and poor recovery prognosis^1,2^.

Although the toxic effects of cigarette smoking have been widely investigated, the exact mechanisms behind their negative impact in wound healing are not fully understood, which is why the need for new and reliable *in vivo* models is urgent. In this work we were able to validate the zebrafish as a bioassay system to study the toxic effects of an aqueous cigarette smoke extract. This extract was prepared from the combustion of three cigarettes of a popularly commercially available tobacco brand as we described before^15^, but, in this case, highly diluted in fish water or larvae growing medium. This approach is similar to the one used recently by other groups^38,47^, and has the advantage of allowing the rapid exposure of the test system to the freshly obtained extract, unlike other methods that use filters or DMSO-supplemented dilutions from the collected particulate^35,36^. This is important, as it is well described that fresh cigarette smoke consists of a particulate solid phase, known as cigarette tar, that contains very high concentrations of stable free radicals, but also of a mixture of numerous chemicals, reactive oxygen species (ROS), volatile organic compounds and a great variety of carbon- and oxygen-centered reactive radicals of short lifetimes found in the smoke gas phase^32^. In order to better mimic the smoking condition in humans, smoking rate as well as the number of cigarettes were chosen to approximate the conditions defined as light smoking, which nevertheless has already been correlated to substantial health risks^48^.

Our results show that CSE is highly noxious for zebrafish, and even low dilutions such as 0.25% CSE were toxic enough to elicit major developmental changes in the zebrafish embryos and larvae, while concentrations above 0.5% CSE were found to be lethal after 96 hours of exposure (Fig. 1). Yet, it is important to note, that the extraction method described in this paper is likely to exclude most lipophilic molecules, thus representing only a fraction of the repertoire of toxic compounds of cigarette smoke and, therefore, further studies should address this issue in more detail. In this regard, we intended to validate the consistency of CSE preparation with our experimental setup by monitoring pH and tar-content of the CSE and CSE-dilutions used (Supp. Fig.). However, while the results obtained support the reproducibility of the protocol, a proper chemical analysis of the sample would be necessary to determine the exact composition of the obtained aqueous CSE, and hence the identity of the toxins the fish are being exposed to. Also, it would be interesting to investigate if CSE-toxicity could be mitigated by using a buffer that compensates the acidification of the medium caused by CSE.

However, our observations regarding the effect of CSE on zebrafish development (Fig. 2), mostly agree with previous results recently reported by other groups. For instance, decreased heart beat and circulation defects, as well as heart malformation, pericardial edema formation and disrupted angiogenesis^35,36,38^. Additionally, other hallmarks of developmental retardation such as impaired hatching and motion^37,39^ have all been described to have a dose-dependent correlation with CSE-exposure. Furthermore, these observations correlate with the most significant smoking-induced pathologies reported in unborn children such as intrauterine growth retardation, fetal heart defects, neurological development, and congenital malformations^49^. Hence, the evidence gathered in all these studies strongly supports the use of the zebrafish as a reliable model to study the adverse effects of smoking during pregnancy.

Because angiogenesis plays a key role in wound healing and regeneration, and it has been found to be altered in smoking-related impaired wound healing^50,51^, we decided to investigate the effect of CSE in the angiogenic process observed during blood vessel development. For this, we focused in the formation of the intersegmental vessels, which have been proposed and validated as an advantageous zebrafish-based angiogenesis assay^34,52,53^. After exposure to CSE a significant impairment in the ISV development caused by CSE exposure was observed (Fig. 3), shown by the inability of ISVs to elongate and anastomose to neighboring ISVs when zebrafish embryos were treated with over 0.5% CSE. While this could be a side effect of the developmental retardation caused by CSE (Fig. 2A,B), it was interesting to note that a lower CSE-concentration, that did affect developmental timing (0.25% CSE, Fig. 2A), did not influence ISV development significantly (0.25% CSE, Fig. 3). Nevertheless, our result agrees with the observations reported using a CAM-assay model, where the construction and integrity of the secondary and tertiary vasculature was compromised in the presence of CSC and TPM^30^.

After studying the toxicity and effects of CSE on embryonic development, we then decided to investigate the effects of CSE on the regenerative capacity of adult zebrafish, and explore the possibility of establishing a new *in vivo* model to study the impaired regeneration capacity described in smokers. Even though, it has been shown that the regeneration process in zebrafish larvae follows similar cellular and molecular mechanisms compared to adult regeneration^54^, adult zebrafish tissues are considered to be more complex, as they are built out of different cell types with specific structure and function, with both cell state plasticity and epigenetic programming. Therefore, the use of adult zebrafish may represent a more accurate preclinical regeneration model than its larval stages. In this regard, adult caudal fin regeneration is one of the better established and best understood regeneration *in vivo* models available to date^40,55^.

In order to evaluate the effect of CSE in caudal fin regeneration, adult zebrafish survival was evaluated after exposure to serial dilutions of CSE for a period of time of 20 consecutive days. We discovered that CSE dilutions over 0.5% CSE were lethal for the zebrafish, which would correspond to 0.6 ·10-3 cigarettes/ml (Fig. 4A), and would be consistent to what Progatzky *et al*. reported as lethal for adult zebrafish after 24 hours (1.0·10-3 cigarettes/ml)^47^. Unexpectedly, adult fish were significantly more sensitive than larval stage animals. A detailed study of the molecular mechanisms that protect fish at early stages, could not only help to understand the nature of cigarette smoke toxicity, but also be used to develop new strategies to decrease its toxicity for smokers.

Our results showed that, after amputation, the regenerated area of the fin was significantly smaller when fish were exposed to CSE (Fig. 4B,C), resembling the impaired wound healing outcome observed in smokers. Furthermore, male fish as well as aged fish appeared to be the most susceptible groups to CSE-exposure (Fig. 5C,F), given the decreased speed at which they were able to regrow the lost tissue. Moreover, if the averaged final regenerated area of each treated group is placed in relation to the averaged regenerated area of their respective control group, we see that young fish (31.2 ± 1.6% reduction in growth) and female (33.7 ± 1.5% reduction in growth) zebrafish are less affected than male (35.5 ± 1.8% reduction in growth) or aged fish (37.7 ± 1.2% reduction in growth). However, statistical analysis (one-way Anova) in this case did not reveal a significant difference among the groups, partly because of the heterogeneity in the number of individuals belonging to each group, and partly because of lack of a proper normalization constraint in our experimental setup (*e*.*g*. fin area of each fish before amputation). According to the literature, there is no difference between the male and female zebrafish caudal fin regenerative potential^56^. On the other hand, aging has been reported to affect the ability of zebrafish to restore lost tissue^57^. In this regard, while cigarette smoke is well-known to accelerate skin aging^3^, sex hormones, such as female estrogens, have been shown to play a role in age-related wound-healing deficits^5^. Further experiments would be necessary to elucidate the exact mechanisms affected by CSE in each of these target groups, as well as whether age affects female and male zebrafish equally.

In order to search for a physiological mechanism that could partially explain the detrimental effects of CSE in caudal fin regeneration, we investigated whether exposure to CSE could have had an effect in angiogenesis of the adult zebrafish, and consequently, in the regeneration of the fin. Angiogenesis is one of the key mediators for fin regeneration^58^, and moreover, CSE-induced angiogenesis impairment has already been shown in other *in vitro*^59,60^ and *in vivo* models^27,30,61^. Furthermore, CSE has an effect on zebrafish angiogenesis at larval stages (this study). Yet, surprisingly, CSE exposure had no effect on the re-vascularization capacity of the exposed zebrafish. There are two additional mechanisms, which could potentially be involved in the regeneration impairment as a result of CSE exposure. First, we and others have previously shown that CSE is highly oxidative and, what is more, that by targeting CSE toxicity with antioxidant strategies it is possible to ameliorate the detrimental effects of CSE in *in vitro* approaches^17,18,62^ and in zebrafish larval development^63,64^. While reactive oxidative species (ROS) are required for proper wound healing as signaling molecules and antibacterial agents^65,66^, sustained oxidative stress and increased presence of ROS triggers an abnormal inflammatory response, which has been widely reported in smokers^67–69^. Secondly, and directly related to this, it has been recently shown that cigarette smoke triggers aberrant inflammatory responses in the adult zebrafish gills. The authors reported dramatic morphological changes, such as lamellar fusion and epithelial cell hyperplasia, as well as a reduction in the number of neutrophils in the zebrafish gills following acute exposure to CSE^47^. Hence, further studies should address whether the decreased regeneration potential is triggered by inflammatory mechanisms and whether it could be rescued by the use of antioxidative molecules. Additionally, further studies should investigate the effect of CSE in other processes that have been partially addressed in other models, such as proliferation^70^, apoptosis rate^71^, correct chondrogenesis, as well as callus formation and bone differentiation^72^. Finally, the potential of the zebrafish model presented here could be maximally exploited, if other reporter transgenic zebrafish lines were used to track cell-type specific migration and differentiation, as well as to address faithful tissue restoration *in vivo* and over the entire regeneration window of time, in order to understand how exactly exposure to cigarette smoke affects regeneration and diminish its toxic effects.

## Conclusion

In this work, we describe the use of the zebrafish caudal fin as model to study the toxic effect of cigarette smoke exposure and as a new tool to further study the mechanisms involved in CSE-impaired regeneration for the first time. We believe its advantages above other *in vivo* models make it a valuable tool for the understanding the spectrum of wound healing mechanisms affected by cigarette smoke, as well as in the development and evaluation of strategies that mitigate the damage induced in human tissues. Finally, since our approach is based on an aqueous solution in which a gas is dissolved, our model could also be used to study other air pollutants such as industrial residues or combustion products, which further affect human health worldwide.

## Electronic supplementary material

Supplementary information

## References

[1] World Health Organization. WHO report on the global tobacco epidemic, 2011: warning about the dangers of tobacco. Geneva, Switzerland http://www.who.int/tobacco/global_report/2011/en/ (2011).

[2] World Health Organization. WHO global report on trends in prevalence of tobacco smoking 2000–2025. Geneva, Switzerland http://www.who.int/tobacco/publications/surveillance/reportontrendstobaccosmoking/en/index4.html (2015).

[3] Morita A (2007). Tobacco smoke causes premature skin aging. Journal of dermatological science.

[4] Bagaitkar J, Demuth DR, Scott DA (2008). Tobacco use increases susceptibility to bacterial infection. Tobacco induced diseases.

[5] Guo S, Dipietro LA (2010). Factors affecting wound healing. Journal of dental research.

[6] Sorensen LT (2012). Wound healing and infection in surgery: the pathophysiological impact of smoking, smoking cessation, and nicotine replacement therapy: a systematic review. Annals of surgery.

[7] Goltsman D, Munabi NC, Ascherman JA (2017). The Association between Smoking and Plastic Surgery Outcomes in 40,465 Patients: An Analysis of the American College of Surgeons National Surgical Quality Improvement Program Data Sets. Plastic and reconstructive surgery.

[8] Gaston MS, Simpson AH (2007). Inhibition of fracture healing. The Journal of bone and joint surgery. British volume.

[9] Wright E (2016). Effect of Smoking on Joint Replacement Outcomes: Opportunities for Improvement Through Preoperative Smoking Cessation. Instructional course lectures.

[10] Mulligan RP (2018). Preoperative Risk Factors for Complications in Elective Ankle and Hindfoot Reconstruction. Foot & ankle specialist.

[11] Lenselink E, Holloway S, Eefting D (2017). Outcomes after foot surgery in people with a diabetic foot ulcer and a 12-month follow-up. Journal of wound care.

[12] Lane CA, Selleck C, Chen Y, Tang Y (2016). The Impact of Smoking and Smoking Cessation on Wound Healing in Spinal Cord-Injured Patients With Pressure Injuries: A Retrospective Comparison Cohort Study. Journal of wound, ostomy, and continence nursing: official publication of The Wound, Ostomy and Continence Nurses Society.

[13] Suckow BD, Stone DH (2015). Vascular surgery institutional-based quality and performance measures for the care of patients with critical limb ischemia. Seminars in vascular surgery.

[14] Truntzer J, Vopat B, Feldstein M, Matityahu A (2015). Smoking cessation and bone healing: optimal cessation timing. European journal of orthopaedic surgery & traumatology: orthopedie traumatologie.

[15] Wahl EA, Schenck TL, Machens HG, Egana JT (2016). Acute stimulation of mesenchymal stem cells with cigarette smoke extract affects their migration, differentiation, and paracrine potential. Sci Rep.

[16] Sreekumar, V. *et al*. Resveratrol protects primary cilia integrity of human mesenchymal stem cells from cigarette smoke to improve osteogenic differentiation *in vitro*. *Archives of toxicology*, 10.1007/s00204-017-2149-9 (2017). 10.1007/s00204-017-2149-9 29264620

[17] Braun KF (2011). Quercetin protects primary human osteoblasts exposed to cigarette smoke through activation of the antioxidative enzymes HO-1 and SOD-1. TheScientificWorldJournal.

[18] Holzer N (2012). Green tea protects human osteoblasts from cigarette smoke-induced injury: possible clinical implication. Langenbeck’s archives of surgery.

[19] Di Stefano R (2010). Smoking and endothelial progenitor cells: a revision of literature. Current pharmaceutical design.

[20] Gornati R (2013). Protein carbonylation in human endothelial cells exposed to cigarette smoke extract. Toxicology letters.

[21] Messner B (2012). Apoptosis and necrosis: two different outcomes of cigarette smoke condensate-induced endothelial cell death. Cell death & disease.

[22] Egawa M, Kohno Y, Kumano Y (1999). Oxidative effects of cigarette smoke on the human skin. International journal of cosmetic science.

[23] Yang GY, Zhang CL, Liu XC, Qian G, Deng DQ (2013). Effects of cigarette smoke extracts on the growth and senescence of skin fibroblasts *in vitro*. International journal of biological sciences.

[24] Santiago HA, Zamarioli A, Sousa Neto MD, Volpon JB (2017). Exposure to Secondhand Smoke Impairs Fracture Healing in Rats. Clinical orthopaedics and related research.

[25] Franck FC (2017). Impact of resveratrol on bone repair in rats exposed to cigarette smoke inhalation: histomorphometric and bone-related gene expression analysis. International journal of oral and maxillofacial surgery.

[26] Franco GR (2013). Effects of chronic passive smoking on the regeneration of rat femoral defects filled with hydroxyapatite and stimulated by laser therapy. Injury.

[27] Ejaz S, Ashraf M, Nawaz M, Lim CW (2009). Total particulate matter and wound healing: an *in vivo* study with histological insights. Biomedical and environmental sciences: BES.

[28] Ueha R (2016). Cigarette Smoke Delays Regeneration of the Olfactory Epithelium in Mice. Neurotoxicity research.

[29] Trombitas V, Nagy A, Berce C, Tabaran F, Albu S (2016). Effect of Cigarette Smoke on Wound Healing of the Septal Mucosa of the Rat. BioMed research international.

[30] Ejaz S (2009). Cigarette smoke condensate and total particulate matter severely disrupts physiological angiogenesis. Food and chemical toxicology: an international journal published for the British Industrial Biological Research Association.

[31] Borgerding, M. & Klus, H. Analysis of complex mixtures–cigarette smoke. *Experimental and toxicologic pathology: official journal of the Gesellschaft fur Toxikologische Pathologi*e **57**Suppl 1, 43–73 (2005). 10.1016/j.etp.2005.05.010 16092717

[32] Valavanidis A, Vlachogianni T, Fiotakis K (2009). Tobacco smoke: involvement of reactive oxygen species and stable free radicals in mechanisms of oxidative damage, carcinogenesis and synergistic effects with other respirable particles. International journal of environmental research and public health.

[33] Talhout R (2011). Hazardous compounds in tobacco smoke. International journal of environmental research and public health.

[34] Chavez MN, Aedo G, Fierro FA, Allende ML, Egana JT (2016). Zebrafish as an Emerging Model Organism to Study Angiogenesis in Development and Regeneration. Frontiers in physiology.

[35] Ellis LD, Soo EC, Achenbach JC, Morash MG, Soanes KH (2014). Use of the zebrafish larvae as a model to study cigarette smoke condensate toxicity. PLoS One.

[36] Massarsky A (2015). Teratogenic, bioenergetic, and behavioral effects of exposure to total particulate matter on early development of zebrafish (Danio rerio) are not mimicked by nicotine. Neurotoxicology and teratology.

[37] Hammer TR, Fischer K, Mueller M, Hoefer D (2011). Effects of cigarette smoke residues from textiles on fibroblasts, neurocytes and zebrafish embryos and nicotine permeation through human skin. International journal of hygiene and environmental health.

[38] Palpant NJ, Hofsteen P, Pabon L, Reinecke H, Murry CE (2015). Cardiac development in zebrafish and human embryonic stem cells is inhibited by exposure to tobacco cigarettes and e-cigarettes. PLoS One.

[39] Folkesson M (2016). Differences in cardiovascular toxicities associated with cigarette smoking and snuff use revealed using novel zebrafish models. Biology open.

[40] Pfefferli C, Jazwinska A (2015). The art of fin regeneration in zebrafish. Regeneration.

[41] Lawson ND, Weinstein BM (2002). *In vivo* imaging of embryonic vascular development using transgenic zebrafish. Dev Biol.

[42] Perry, S., Ekker, M., Farrell, A., Brauner, C. Fish Physiology: Zebrafish. Volume 29. p. 252. (Academic Press, 2010).

[43] Kimmel CB, Ballard WW, Kimmel SR, Ullmann B, Schilling TF (1995). Stages of Embryonic Development of the Zebrafish. Dev Dyn.

[44] Bliss CI (1934). The Method of Probits. Science.

[45] Azevedo AS, Grotek B, Jacinto A, Weidinger G, Saude L (2011). The regenerative capacity of the zebrafish caudal fin is not affected by repeated amputations. PLoS One.

[46] Schindelin J, Rueden CT, Hiner MC, Eliceiri KW (2015). The ImageJ ecosystem: An open platform for biomedical image analysis. Molecular reproduction and development.

[47] Progatzky F, Cook HT, Lamb JR, Bugeon L, Dallman MJ (2016). Mucosal inflammation at the respiratory interface: a zebrafish model. American journal of physiology. Lung cellular and molecular physiology.

[48] Schane RE, Ling PM, Glantz SA (2010). Health effects of light and intermittent smoking: a review. Circulation.

[49] Mund M, Louwen F, Klingelhoefer D, Gerber A (2013). Smoking and pregnancy–a review on the first major environmental risk factor of the unborn. International journal of environmental research and public health.

[50] Anderson K, Hamm RL (2012). Factors That Impair Wound Healing. The journal of the American College of Clinical Wound Specialists.

[51] McDaniel, J. C. & Browning, K. K. Smoking, chronic wound healing, and implications for evidence-based practice. *Journal of wound*, *ostomy*, *and continence nursing: official publication of The Wound*, *Ostomy and Continence Nurses Society* **41**, 415–423; quiz E411–412, 10.1097/WON.0000000000000057 (2014). 10.1097/WON.0000000000000057 PMC4241583 25188797

[52] Delov V, Muth-Kohne E, Schafers C, Fenske M (2014). Transgenic fluorescent zebrafish Tg(fli1: EGFP)y(1) for the identification of vasotoxicity within the zFET. Aquatic toxicology.

[53] Garcia-Caballero M, Quesada AR, Medina MA, Mari-Beffa M (2018). Fishing anti(lymph)angiogenic drugs with zebrafish. Drug discovery today.

[54] Kawakami A, Fukazawa T, Takeda H (2004). Early fin primordia of zebrafish larvae regenerate by a similar growth control mechanism with adult regeneration. Developmental dynamics: an official publication of the American Association of Anatomists.

[55] Gemberling M, Bailey TJ, Hyde DR, Poss KD (2013). The zebrafish as a model for complex tissue regeneration. Trends in genetics: TIG.

[56] Nachtrab G, Czerwinski M, Poss KD (2011). Sexually dimorphic fin regeneration in zebrafish controlled by androgen/GSK3 signaling. Current biology: CB.

[57] Shao J (2011). Tissue regeneration after injury in adult zebrafish: the regenerative potential of the caudal fin. Developmental dynamics: an official publication of the American Association of Anatomists.

[58] Bayliss PE (2006). Chemical modulation of receptor signaling inhibits regenerative angiogenesis in adult zebrafish. Nature chemical biology.

[59] Michaud SE, Dussault S, Groleau J, Haddad P, Rivard A (2006). Cigarette smoke exposure impairs VEGF-induced endothelial cell migration: role of NO and reactive oxygen species. Journal of molecular and cellular cardiology.

[60] Edirisinghe I (2010). Cigarette-smoke-induced oxidative/nitrosative stress impairs VEGF- and fluid-shear-stress-mediated signaling in endothelial cells. Antioxidants & redox signaling.

[61] Michaud SE, Menard C, Guy LG, Gennaro G, Rivard A (2003). Inhibition of hypoxia-induced angiogenesis by cigarette smoke exposure: impairment of the HIF-1alpha/VEGF pathway. FASEB journal: official publication of the Federation of American Societies for Experimental Biology.

[62] Tan X (2014). Protective effect of luteolin on cigarette smoke extract-induced cellular toxicity and apoptosis in normal human bronchial epithelial cells via the Nrf2 pathway. Oncology reports.

[63] Massarsky A (2016). AHR2 morpholino knockdown reduces the toxicity of total particulate matter to zebrafish embryos. Toxicology and applied pharmacology.

[64] Massarsky A, Prasad GL, Di Giulio RT (2018). Total particulate matter from cigarette smoke disrupts vascular development in zebrafish brain (Danio rerio). Toxicology and applied pharmacology.

[65] auf dem Keller U, Kumin A, Braun S, Werner S (2006). Reactive oxygen species and their detoxification in healing skin wounds. The journal of investigative dermatology. Symposium proceedings.

[66] Dunnill C (2017). Reactive oxygen species (ROS) and wound healing: the functional role of ROS and emerging ROS-modulating technologies for augmentation of the healing process. International wound journal.

[67] Moreira DM (2015). Smoking Is Associated with Acute and Chronic ProstaticInflammation: Results from the REDUCE Study. Cancer prevention research.

[68] Crotty Alexander LE, Shin S, Hwang JH (2015). Inflammatory Diseases of the Lung Induced by Conventional Cigarette Smoke: A Review. Chest.

[69] Luetragoon T (2017). Interaction among smoking status, single nucleotide polymorphisms and markers of systemic inflammation in healthy individuals. Immunology.

[70] Gill CS, Sandell LJ, El-Zawawy HB, Wright RW (2006). Effects of cigarette smoking on early medial collateral ligament healing in a mouse model. Journal of orthopaedic research: official publication of the Orthopaedic Research Society.

[71] Martin JW, Mousa SS, Shaker O, Mousa SA (2009). The multiple faces of nicotine and its implications in tissue and wound repair. Experimental dermatology.

[72] El-Zawawy HB, Gill CS, Wright RW, Sandell LJ (2006). Smoking delays chondrogenesis in a mouse model of closed tibial fracture healing. Journal of orthopaedic research: official publication of the Orthopaedic Research Society.

