# Supplementary material for: A Novel In Vivo Model to Study Impaired Tissue Regeneration Mediated by Cigarette Smoke
Source: Sci Rep. 2018 Jul 19;8:10926. doi: 10.1038/s41598-018-28687-1 (PMC6053433; doi:10.1038/s41598-018-28687-1)

A Novel *In Vivo* Model to Study Impaired Tissue Regeneration Mediated by Cigarette Smoke.

Marjorie Alvarez[1,†], Myra N. Chávez[1,2,†], Miguel Miranda[1,3,†], Geraldine Aedo[1], Miguel L. Allende[1*], José T. Egaña[4*].

[1]FONDAP Center for Genome Regulation, Facultad de Ciencias, Universidad de Chile. [2]Advanced Center for Chronic Disease (ACCDiS) & Center for Molecular Studies of the Cell (CEMC), Facultad de Ciencias Químicas y Farmacéuticas & Facultad de Medicina, Universidad de Chile, Santiago, Chile. [3]Facultad de Medicina Veterinaria y Agronomía, Universidad de las Américas, Santiago, Chile. [4]Institute for Biological and Medical Engineering, Schools of Engineering, Medicine and Biological Sciences, Pontifícia Universidad Católica de Chile, Santiago, Chile.

[†]***Authors contributed equally to this work***

[*]***Corresponding authors***

Correspondence to joegana@uc.cl, mallende@uchile.cl.

SUPPLEMENTARY INFORMATION

Tar-content in the CSE (Supp. Fig. A)

Tar-content of the CSE was determined by optical density (OD_{320}). For this, CSE and CSE-dilutions (0.25%, 0.50%, 0.75%, 1.0%, 1.5%, and 2.0%) were freshly prepared in 3 ml E3-medium in duplicate as described in the materials and methods section. Then, optical density measurement was performed at 320 nm in 1 ml quartz cuvettes with a multimode spectrophotometer (Jenway 6705 UV/Vis. Spectrophotometer, Cole-Parmer, Staffordshire, UK). The results were obtained from the three independent CSE-preparations and their respective dilution series, and compared to E3-medium. *$p<0.05$.

pH measurements (Supp. Fig. B)

The pH of the CSE and its serial dilutions was determined using a previously calibrated benchtop pH-meter (calibration range 4.01-7.01, HI2020-02 Edge®, Hanna Instruments, Rhode Island, USA). For this, freshly prepared CSE was diluted in 10 ml E3-medium to the final concentrations of 0.25%, 0.50%, 0.75%, 1.0%, 1.5%, and 2.0% in duplicate. The results were obtained from three independent CSE-preparations and their respective dilution series, and compared to E3-medium. *$p<0.05$.

SUPPLEMENTARY FIGURE

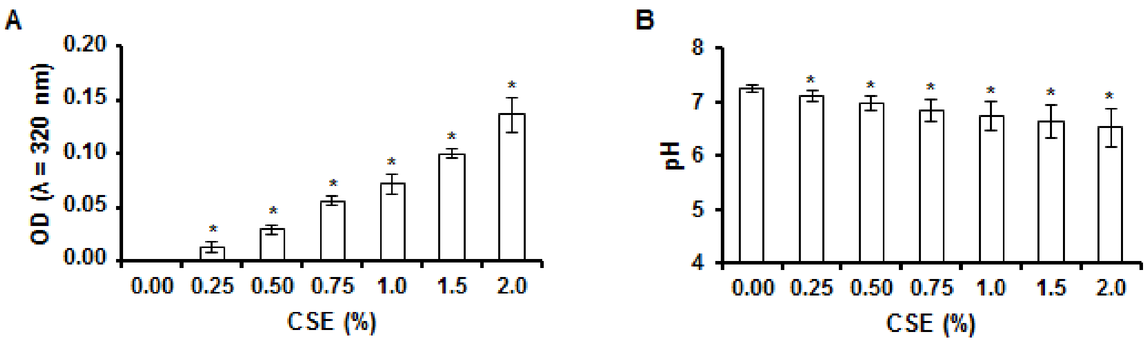

Supplement: Supplementary file 1 — Supplementary information [file 41598_2018_28687_MOESM1_ESM.pdf]
